# An isofagomine analogue with an amidine group in the 1,6-position

**DOI:** 10.1098/rsos.241877

**Published:** 2025-02-19

**Authors:** I. Caroline Vaaland Holmgard, Idd Andrea Christensen, Ljupcho Pejov, Monika Moreń, Nikolai Westlund, Tereza Cristina Santos Evangelista, Magne O. Sydnes, Finn L. Aachmann, Óscar López, Emil Lindbäck

**Affiliations:** ^1^ Department of Chemistry, Bioscience and Environmental Engineering, Faculty of Science and Technology, University of Stavanger, Stavanger, Norway; ^2^ Norwegian Biopolymer Laboratory (NOBIPOL), Department of Biotechnology and Food Science, NTNU Norwegian University of Science and Technology, Trondheim, Norway; ^3^ Institute of Chemistry, Faculty of Natural Sciences and Mathematics, SS Cyril and Methodius University, Skopje, North Macedonia; ^4^ Environmental and Resource Management Program, The Polytechnic School, Ira A. Fulton Schools of Engineering, Arizona State University, Tempe, AZ, USA; ^5^ Department of Chemistry, Faculty of Science and Technology, University of Bergen, Bergen, Norway; ^6^ Departamento de Química Orgánica, Facultad de Química, Universidad de Sevilla, Seville, Spain

**Keywords:** isofagomine, analogue, amidines, 1,6-position

## Abstract

The synthesis of an isofagomine analogue with an amidine group in the 1,6-position is described. Density functional theory calculations showed that this compound has a remarkably different charge distribution compared with isofagomine. This may explain why the amidine is a poor glycosidase inhibitor (IC_50_ > 50 µM against all tested enzymes) compared with isofagomine.

## 1. Introduction

Glycosidases are a ubiquitous group of enzymes in living organisms, catalysing the hydrolysis of glycosidic bonds during the degradation of polysaccharides and glycoconjugates. Iminosugars represent a group of carbohydrate mimetics that are well known for their frequently high activity as glycosidase inhibitors [1]. This activity has made them attractive as lead compounds for the treatment of a wide spectrum of diseases such as cancer [2], diabetes [3], lysosomal storage disorders [4] and lately COVID-19 [5].

**Figure 1.**
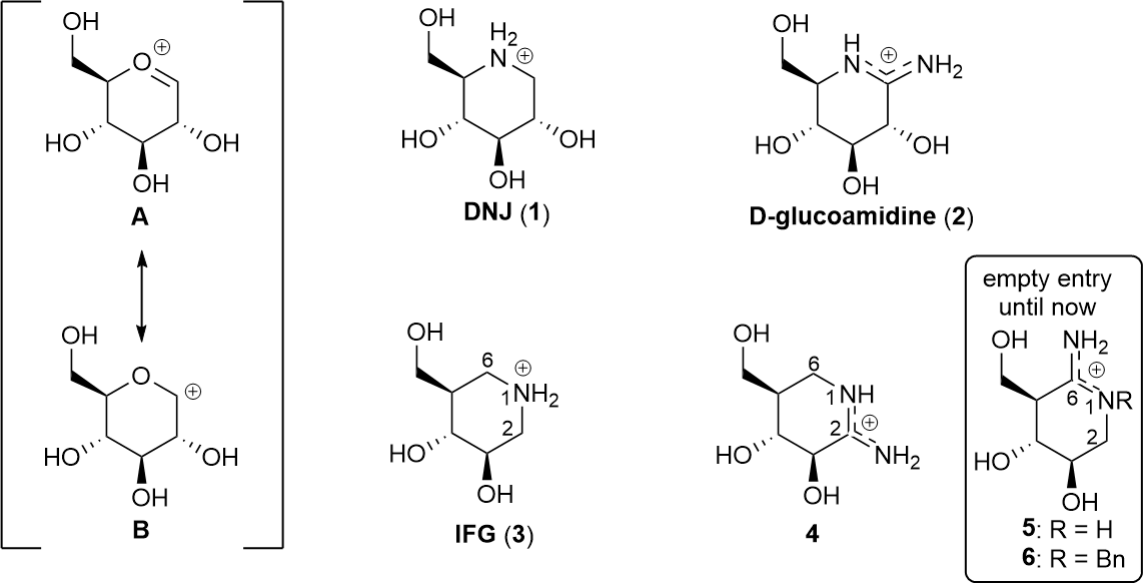
Oxocarbenium ion **A** and its resonance form **B**, and examples of iminosugars and azasugars. Amidines **5** and **6** are the target compounds in this work.

The high glycosidase inhibition activity of basic iminosugars, such as 1-deoxynojirimycin (DNJ) (**1**) (figure 1), which is a mimic of glucose, has been attributed to their charge resemblance to the oxocarbenium ion (**A**)-like transition state in their protonated states. This allows them to interact with the catalytic carboxyl and/or carboxylate groups in the active site of glycosidases through ionic bonds [6]. However, because **1** adopts a chair conformation, it is not a perfect shape mimic of oxocarbenium ion **A** that adopts a more flattened conformation. Therefore, Ganem and co-workers tailored ᴅ-glucoamidine (**2**) (figure 1), which is an analogue of glucose, assumed to mimic both the shape and charge distribution of the glycosidase transition state [7]. This compound, which is a roughly 7900-fold stronger base than **1**, was found to inhibit a broad spectrum of glycosidases [7,8]. The low configurational selectivity of **2** has been argued to indicate that it is not a perfect mimic of the transition state [6].

Azasugars, exemplified by isofagomine (IFG) (**3**) (figure 1) [9], are a group of glycosidase inhibitors related to iminosugars that contain a nitrogen atom in the pseudo anomeric position. The conjugate acids of such compounds resemble the resonance form **B** of the oxocarbenium ion **A** [10]. X-ray crystallographic studies have demonstrated that the attachment of a β-glucopyranosyl group in the 4-position of isofagomine provides an inhibitor that binds to cellulase Cel5A through ionic interactions with the catalytic carboxyl and carboxylate groups in the active site [11]. Insertion of an oxo group in the 2-position of **3** provided a non-basic lactam analogue that is a much weaker glycosidase inhibitor than **3** [12], indicating that a basic nitrogen atom in the anomeric position has an enhancing effect on the glycosidase inhibitory activity. Following this line, compound **4** (figure 1), which is an analogue of isofagomine (**3**), containing a basic amidine group at the 1,2-position, was prepared [13]. This compound was found to exhibit a very different inhibition profile compared with **3** since it is a much stronger jack bean α-mannosidase inhibitor (*K*_i_ = 0.75 µM for **4** [13] and IC_50_ = 250 µM for **3** [14]) and a much weaker almond β-glucosidase inhibitor (*K*_i_ = 450 µM for **4** [13] and *K*_i_ = 0.11 µM for **3** [9]). The significantly different inhibition profiles were proposed to be due to variations in the charge distributions in **3** and **4**, in which density functional theory (DFT) calculations showed that **3** has a positive group charge at NH_2_-1, whereas there is a slightly negative group charge at NH-1 in **4** [13].

Herein, we report the synthesis of the regioisomer of **4**, namely amidine **5** and its *N*-benzyl derivative **6** (figure 1), in addition to their glycosidase inhibition activities on a wide panel of commercially available glycosidase and their distribution of atomic and group charges.

**Scheme 1.**
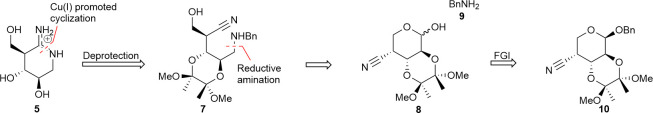
Retrosynthetic analysis of amidine **5**.

The retrosynthetic analysis of amidine **5** is presented in Scheme 1. For the synthesis of amidine **4**, the amidine group was installed by a Cu(I)-promoted intramolecular addition of an amino group to a cyano group [13,15]. Thus, we anticipated that the amidine group in **5** would be installed through Cu(I)-promoted cyclization of amino nitrile precursor **7**. Compound **7** would be synthesized by reductive amination between pyranose **8** and benzylamine (**9**). Pyranose **8** would be obtained when compound **10** [16] undergoes de-*O*-benzylation.

**Scheme 2.**
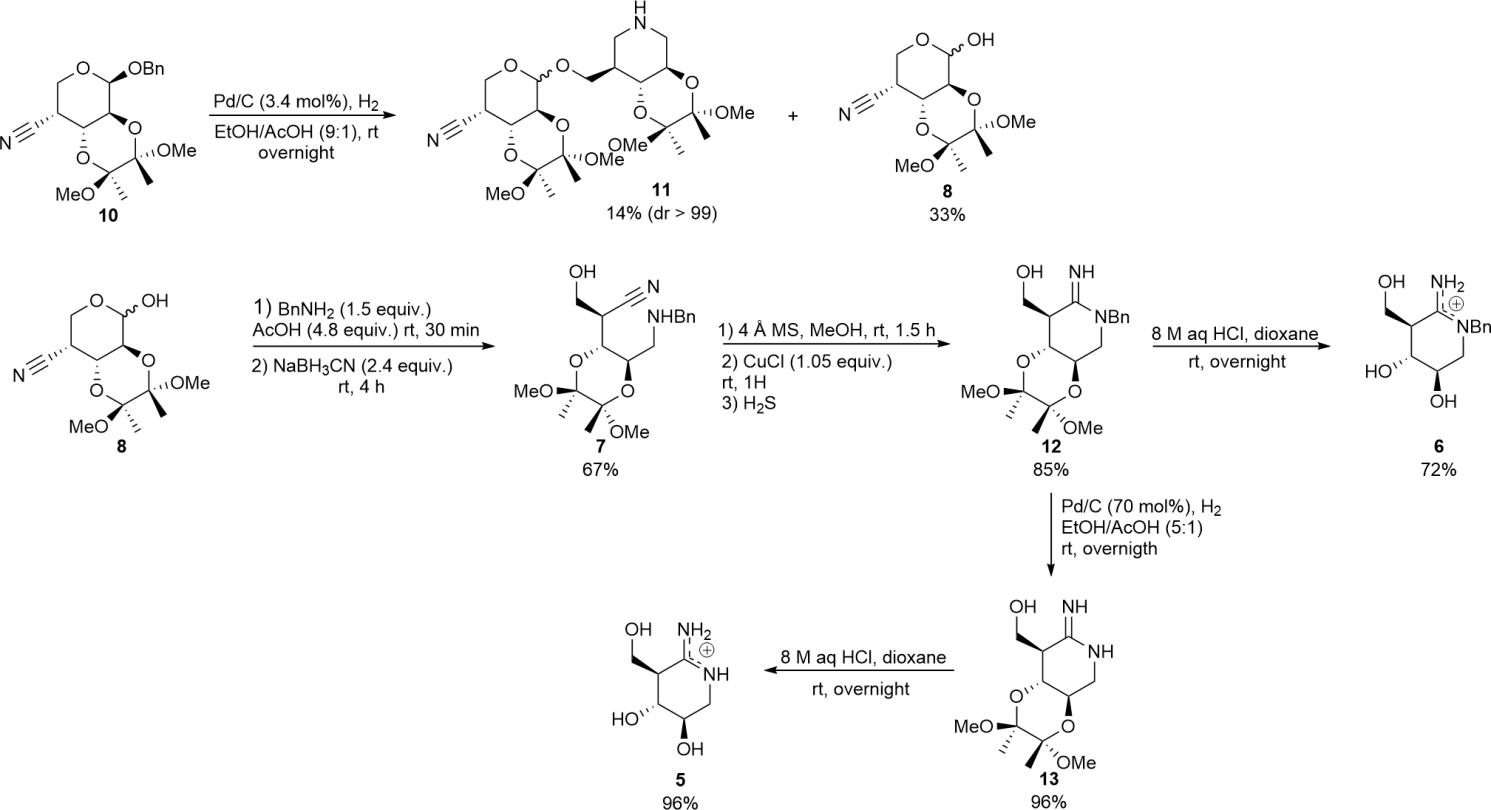
Synthesis of amidines **5** and **6**.

The synthesis of compounds **5** and **6** is presented in Scheme 2. The synthesis commenced with the palladium-catalysed de-*O*-benzylation of benzylarabinopyranoside **10** to generate desired pyranose **8** in 33% yield along with compound **11** in 14% yield and a high diastereomeric ratio (dr) after silica gel flash column chromatography. The configuration of the anomeric carbon of **11** could not be determined because the signal of the anomeric proton overlapped with other signals in the ^1^H nuclear magnetic resonance (NMR) spectra. The formation of **11** was expected to proceed through a de-*O*-benzylation/nitrile hydrogenation/reductive amination sequence of **10** to form a piperidine (i.e. acetal-protected isofagomine) whose primary hydroxyl reacted with **8**. Selective BCl_3_-mediated de-*O*-benzylation of **10** was explored, which unfortunately only gave a reaction on the acetal ring leaving the *O*-benzyl group untouched. Amino nitrile **7** was obtained in 67% yield through reductive amination when **8** was treated with benzylamine in the presence of sodium cyanoborohydride. Copper(I) chloride triggered the cyclization of **7** into *N*-benzylamidine **12** in 85% yield after removing the Cu-ion residues upon precipitation with H_2_S in methanol followed by subsequent silica gel flash column chromatography. The acetal group in compound **12** was removed upon treatment with aqueous HCl solution to furnish amidine **6** in 72% yield. 70 mol% of palladium on carbon under a hydrogen atmosphere was employed to remove the benzyl group from **12** to afford compound **13** whose acetal group was removed to afford amidine **5** in 96% yield when it was treated with an aqueous solution of HCl.

**Figure 2.**
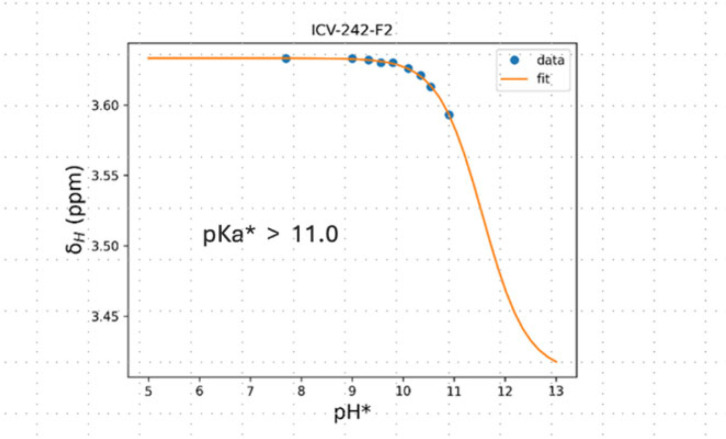
Titration curve of the chemical shifts of 2-Ha versus pH* for amidine **6**.

The p*K*_a_ values for azasugars have been determined by NMR spectroscopy in other studies [17]. This methodology was also applied in this study to determine the p*K*_a_ value of **6**. Specifically, the chemical shifts of the 2-Ha proton were plotted against the pH* (the pH measured in D_2_O using a H_2_O-calibrated pH electrode), which provided an inflection point on the resulting best-fit sigmoidal curve at pH* > 11.0, corresponding to the p*K*_a_* value for **6** (figure 2). This p*K*_a_* value was corrected to p*K*_a_ > 10.6 when a correction factor was applied [18]. The sigmoidal curve is missing points at high pH* values (greater than 11) because **6** started degrading at roughly pH* 10.5. Recording of both a ^1^H NMR and ^13^C HSQC spectra at each titration point allowed us to track the changes in chemical shifts at pH levels higher than where the degradation initially started. The p*K*_a_ of amidine **5** was determined at even lower accuracy than for **6**, because it started degrading at roughly pH* 9.4, where there is no indication of **5** being deprotonated. Presumably, the p*K*_a_ of **5** is higher than that for **6**, because iminosugars armed with aromatic moieties via a CH_2_ group have been found to have lower p*K*_a_ values than the corresponding iminosugars without such *N*-substituents [19]. Additionally, a high p*K*_a_ value of **5** was expected as the p*K*_a_ value of structurally related ᴅ-glucoamidine (**2**) is also high (≥10.5) [7].

The glycosidase inhibitory activities (IC_50_ (µM)) of amidines **5** and **6** on a panel of six glycosidases are presented in table 1 and compared with structurally related isofagomine (**3**) and amidine **4**. Remarkably, even though the structure of amidine **5** is quite similar to those of **3** and **4**, which are powerful inhibitors of β-glucosidase and α-mannosidase, respectively, it only showed a very weak inhibition (IC_50_ > 50 µM) of both of these enzymes. Indeed, amidine **5** is a very poor inhibitor of all enzymes in the panel. The benzyl group on amidine **6** seems to have no positive impact on inhibition potency, as this compound is also a very weak inhibitor for all enzymes tested.

**Table 1.** IC_50_ and *K*_i_ values for **3**–**6**.

	IC_50_ (µM) and *K*_i_ (µM)			
enzyme	3	4	5	6
α-glucosidase^a^, pH 6.8	—	—	IC_50_ > 50	IC_50_ > 50
β-glucosidase^b^, pH 6.8	*K*_i_ = 0.11^g^	*K*_i_ = 450^g^	IC_50_ > 50	IC_50_ > 50
α-galactosidase^c^, pH 6.8	IC_50_ > 2000^h^	NI^g,i^	IC_50_ > 50	IC_50_ > 50
β-galactosidase^d^, pH 6.8	IC_50_ = 270^h^	*K*_i_ = 17 400^g^	IC_50_ > 50	IC_50_ > 50
β-galactosidase^e^, pH 6.8	—	—	IC_50_ > 50	IC_50_ > 50
α-mannosidase^f^, pH 5.6	IC_50_ = 250^h^	*K*_i_ = 0.75^g^	IC_50_ >50	IC_50_ > 50

^a^ From *Saccharomyces cerevisiae*.

^b^ From almonds.

^c^ From green coffee beans.

^d^ From *Aspergillus oryzae*.

^e^ From *Escherichia coli.*

^f^ From jack beans.

^g^ [13].

^h^ [14].

^i^ NI = no inhibition.

**Figure 3.**
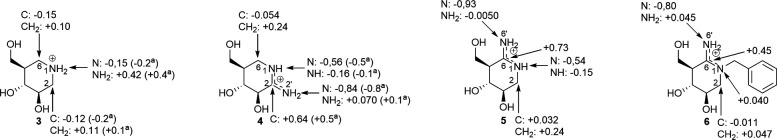
Selected atomic and group charges for **3**–**6**. ^a^From Lindbäck *et al.* [13].

The partial charges of all atoms in **3**–**6** were calculated by the Mulliken, CHelpG and HLY charge partitioning schemes on the basis of DFT electronic densities (electronic supplementary material, tables S1–S4). Selected atomic charges and group charges calculated by the CHelpG scheme for **3**–**6** are presented in figure 3. Our calculated charges agree with previously calculated atomic and group charges for **3** and **4** [13]. Amidines **4**–**6** contain a substantial positive charge at the sp^2^-carbon (i.e. C-2 for **4** and C-6 for **5** and **6**). Interestingly, **4** and **5** have more negative charge at the anomeric nitrogen atom (N-1) than isofagomine (**3**). Additionally, **4** and **5** have a negative group charge at NH-1, in contrast to the positive group charge of NH_2_-1 in isofagomine (**3**). This may explain why **4** and **5** are poor β-glucosidase inhibitors and **3** is a very potent β-glucosidase inhibitor; the negative NH-1 group of **4** and **5** prevents charge–charge interactions with the active site carboxylates, whereas the positive NH_2_-1 group of **3** allows charge–charge interactions with the same residues. Our hypothesis agrees with an earlier study in which two regioisomeric *gluco*-imidazoles were compared for the inhibition of retaining β-glucosidases [20]. The study showed that the compound with the most positive charge on the anomeric carbon displayed the highest activity. However, our hypothesis fails to explain why *N*-benzylamidine **6** is a poor β-glucosidase inhibitor, as it hosts a slight positive charge at N-1. Perhaps the benzyl group connected to N-1 interferes with the charge–charge interactions between N-1 and the active site carboxylates and/or is lacking an interaction site nearby the active site of the enzyme.

## 2. Conclusions

We propose that amidine **5** is a poor β-glucosidase inhibitor because its negatively charged anomeric NH group is unable to establish ionic interactions with the active site carboxylate groups. The fact that **6**, hosting a slightly positive charge on the anomeric nitrogen atom, is also a poor β-glucosidase inhibitor may be due to the Bn group interfering with the formation of charge–charge interactions with the active site carboxylate groups.

## Data Availability

The data supporting this article are included as part of the electronic supplementary material [21].
